# Black Plastic Film Mulching Increases Soil Nitrous Oxide Emissions in Arid Potato Fields

**DOI:** 10.3390/ijerph192316030

**Published:** 2022-11-30

**Authors:** Chaobiao Meng, Jianyu Zhao, Ning Wang, Kaijing Yang, Fengxin Wang

**Affiliations:** 1 Zhejiang Provincial Key Laboratory of Agricultural Resources and Environment, College of Environmental and Resource Sciences, Zhejiang University, Hangzhou 310058, China; 2 Center for Agricultural Water Research in China, China Agricultural University, Beijing 100083, China; 3 China Institute of Water Resources and Hydropower Research, Beijing 100048, China

**Keywords:** nitrous oxide emission, plastic film mulch, *amoA*-*AOB*

## Abstract

Black plastic film mulching is a common practice for potato production in the arid area of Northwest China. Many studies have reported the significant positive effect of black plastic film mulch on potato harvest, while the effect of black plastic film mulch treatment on soil nitrous oxide (N_2_O) emissions is still unclear. As a consequence, this study aimed to examine the effect of black plastic film mulch treatment on N_2_O emission from arid upland potato fields. With the static chamber-gas chromatography method, soil N_2_O emissions were measured. The results showed that black plastic film mulching treatment significantly increased cumulative soil N_2_O emissions by 21–26% compared with non-mulched treatment. Cumulative N_2_O emission positively correlated with soil temperature, soil moisture, soil CO_2_ concentration, and *amoA*-*AOB* abundance. This study indicated that black plastic film mulching, mainly through increasing soil temperature and soil moisture, increasing soil carbon dioxide (CO_2_) concentration, and promoting the abundance of nitrification-related functional gene of *amoA*-*AOB*, regulated N_2_O emissions. This study also highlighted that the specific soil environment under black plastic film mulch is conducive to N_2_O emissions and lay the foundation for settling the contradiction between food production and greenhouse gas mitigation in upland soils. The negative effects of black plastic film mulching on the environment should be considered in future applications in food production.

## 1. Introduction

Nitrous oxide (N_2_O), with 296 times the global warming potential of carbon dioxide within the 100-year time frame [1], has increased by 122% of the pre-industrial level to 329.9 ± 0.1 ppb [2]. Agricultural N_2_O emission is a tremendous global concern, given that agricultural soils contribute 60% of the global anthropogenic N_2_O emissions [3,4]. Potato is the fourth staple food with a 19.3 Mha harvest area worldwide [5]. Potato fields can potentially contribute to global N_2_O emissions.

Black plastic film mulching, a soil temperature retention soil surface management practice, has been widely used [6,7]. Mulching treatment can significantly enhance potato growth and harvest. One of our previous studies indicated that black plastic film mulch could enhance potato harvest by 6–30% [8], while few studies have paid attention to the effect of mulching on N_2_O emissions in arid and semi-arid potato fields. Soils covered with black plastic film mulch had higher soil temperatures compared to non-mulched soils in potato fields [9]. Mulching has been reported to significantly increase N_2_O emissions in the radish field owing to higher temperatures [10]. An exponential relationship between N_2_O flux and the soil temperature has been observed at 0 to 30 °C, while warming can also inhibit nitrification-induced N_2_O emission when soil temperature is over 30 °C [11]. Additionally, extremely high soil surface temperature (>30 °C) under plastic film mulching has been recorded from 10:00 a.m. to 6:00 p.m. during sprout development and vegetative growth stages in an arid potato field experiment [12]. Plastic film mulching could also reduce cumulative N_2_O emissions, which may contribute to the unsuitable soil temperature for N_2_O emission under mulching [13]. The uncertain effect of black plastic film mulching on N_2_O emission makes it urgent to investigate how this agronomic practice affects N_2_O emission in arid potato fields.

Compared to non-mulched treatment, black plastic film mulching could only insignificantly reduce N_2_O emissions owing to lower soil moisture if there is no irrigation during cultivation [10], while black plastic film mulching is always applied with drip irrigation in arid and semi-arid potato filed. In the black plastic film mulching–drip irrigation system, black plastic film mulch could maintain soil moisture at the 0–100 cm soil layer by reducing soil evaporation [14,15]. Topsoil moisture under black plastic film mulch was generally higher than that under non-mulched soil during the whole growing period [16,17]. On the other hand, nitrification and denitrification are two important pathways for N_2_O production and were mainly driven by nitrification-related microorganisms (*amoA*-*AOA* and *amoA*-*AOB*) and denitrification-related microorganisms (*nirS* and *nirK*) [18,19]. Soil moisture generally regulates the main microorganisms for N_2_O production. Nitrification can be an important pathway leading to N_2_O production when soils were incubated in 60% WFPS, and *amoA*-*AOB* would play a key role in N_2_O production [20]. Di suggested that soil moisture had a major influence on ammonia-oxidizing and denitrifying microbial communities and then regulating N_2_O emissions [19]. Denitrification-related communities (*nirS* and *nirK*) and *amoA*-*AOB* are reported to be able to grow under very wet soil conditions (130% field capacity) and produce N_2_O emissions [19]. Therefore, with a higher soil moisture condition under black plastic film, both nitrification and denitrification-related microorganisms may both play an important role in N_2_O, or denitrification-related microorganisms will dominate N_2_O emission. While previous studies paid more attention to the abiotic parameters regulating the effect of black plastic film mulch on soil N_2_O emissions, less attention has been paid to N_2_O-related microorganisms under plastic film mulch.

We hypothesized that black plastic film mulching could increase N_2_O emission by increasing soil temperature and moisture, as well as N_2_O-related microorganisms. We also hypothesized that both nitrification- and denitrification-related microorganisms will increase under mulching treatment leading to a higher N_2_O emission. This study aimed to (1) explore the effect of black plastic film mulching on soil N_2_O emission in the arid potato field, and (2) reveal the mechanisms of how black plastic film mulching affects soil N_2_O emission through regulating soil abiotic and biotic parameters.

## 2. Materials and Methods

### 2.1. Field Experimental Design

The field experiments were conducted in 2017 and 2018 at Shiyanghe Experimental Station in Gansu Province, China (37°52′ N, 102°50′ E, 1581 m a.s.l.). The region has a continental temperate climate. The mean annual temperature and precipitation are 8.8 °C and 164 mm, respectively. Daily air temperature and precipitation during the experiment are shown in Figure S1. The soil at the site is a sandy loam soil (9.1% clay, 31.3% silt, and 59.6% sand) with a pH of 8.2, a field capacity of 0.26 cm^3^/cm^3^, a bulk density of 1.56 g/cm^3^ and a total soil porosity of 41%. Other basic soil properties before the experiment are given in Table S1.

The experimental design was a randomized block design with three replicated plots (5 m × 5.6 m) for each treatment. Each plot had seven isosceles trapezoid ridges and each ridge had a length of 5 m, a width of 0.8 m, and a height of 0.3 m. Treatment of soil covered with (MC) or without (CK) black plastic mulch was settled. The black plastic film mulch was high-density and airtight with 0.008 mm in thickness, and covered soils before potato seeds plantation. The plastic film mulch was perforated for the transplantation of potato seeds. All plots were irrigated by an irrigation system as described in our previous studies [8,21]. Briefly, plots were irrigated when soil volumetric moisture content at 20 cm soil depth declined to 70% (*v/v*) of the field capacity [21]. Soil volumetric moisture contents were monitored by moisture sensors (introduced in Section 2.2). The amount of water irrigated for one irrigation event was 21 mm. Plots of MC treatment were irrigated 21 and 19 times in 2017 and 2018, respectively. Plots of CK treatment were irrigated 24 and 21 times in 2017 and 2018, respectively. All plots received the same fertilizer according to local management, and the fertilization strategies and other agronomic practices are shown in Table S2.

### 2.2. Soil Temperature and Moisture Measurement

Soil temperatures at 10 cm soil depth were monitored by thermal sensors (200TS, Irrometer Co., Inc., Riverside, CA, USA), and soil volumetric moisture at 20 cm soil depth was monitored by soil moisture sensors (200SS, Irrometer Co., Inc., Riverside, CA, USA). The data logger (900M, Irrometer Co., Ltd., Riverside, CA, USA) recorded the data every 10 min. The following equation calculated water-filled pore space (WFPS):

(1) $$WFPS=\frac{volumetric\;moisture\;content}{total\;soil\;porosity}$$

where total soil porosity is 41%.

### 2.3. N_2_O Flux Sampling

Fluxes of N_2_O were measured by a static opaque chamber-gas chromatography method (Supplementary Method). Briefly, a stainless-steel frame was inserted 5 cm into the soil of one ridge in each plot and included soil and two crops. An opaque polymethyl methacrylate chamber was placed on the pre-installed stainless-steel frames. Five gas samples were collected to calculate N_2_O flux around 9 a.m. after chamber closure, at a time interval of 4 min about every 5 days. Gas samples were then kept in pre-vacuumed plastic gasbags (Dalian Pulaite gas packing Co., Ltd., Dalian, China) and analyzed for the N_2_O concentration using a gas chromatograph (GC-2014 series, Shimadzu (China) Co., Ltd., Beijing, China) equipped with an electron capture detector (ECD). The column (SS-2 m × 4 mm Porapak Q (80/100)) and ECD temperatures were maintained at 60 °C and 300 °C, respectively.

Diurnal N_2_O flux samples were collected every 4–6 h over three days at early, middle, and later growth stages in both experimental years to identify the effect of MC treatment on N_2_O flux by alternating soil temperature (Supplementary Method). Flux samples between two irrigation events were also collected every day to identify the relationship between N_2_O flux and soil moisture (Supplementary Method).

Fluxes were calculated with linear function from the change of gas concentration in the chamber during the sampling period by the following equation [21]:

(2) $$F=H\frac{mp}{R\left(273+T\right)}\frac{\partial C}{\partial t}$$

where F is the N_2_O flux (μg m^−2^ min^−1^); H is the height of chamber (m); m is the molecular weight of N_2_O (g mol^−1^); p is the atmospheric pressure (kPa); R is the value of the universal constant; T is the air temperature in the chamber (°C); ∂C/∂t is the change of gas concentration in the chamber during the sampling period (μ mol min^−1^).

Cumulative N_2_O emissions were calculated by trapezoidal integration [22].

### 2.4. Soil Gas (CO_2_ and N_2_O) Sampling

A soil–air equilibration sampler was installed vertically in the soil to a soil depth of 15–20 cm (Supplementary Method and Figure S2). We extracted 20 mL of gas samples between 10:00 a.m. and 10:30 a.m. after soil N_2_O flux sampling, using a syringe through a three-way stopcock connecting with the silicon tube. Gas samples were then kept in pre-vacuumed plastic gasbags (Dalian Pulaite gas packing Co., Ltd., Dalian, China) and analyzed for the N_2_O concentration using a gas chromatograph. The three-way stopcock of the sampler was closed on non-sampling days to avoid the connection of soil air to atmospheric air.

### 2.5. DNA Extraction and Quantitative PCR

Soil samples were collected at the harvest time in 2018 and stored at −80 °C for subsequent DNA extraction and quantitative PCR analysis. Soil DNA was extracted using 0.5 g soil according to the manufactory’s protocol with a Fast DNA Spin Kit for Soil (MP Biomedicals, Eschwege, Germany). Copy numbers of *amoA*-*AOA*, *amoA*-*AOB*, *nirS*, and *nirK* genes were determined by quantitative PCR assays. The primers and thermal cycling conditions are given in Table S3.

### 2.6. Statistical Analysis

The effects of black plastic film mulching treatment on cumulative N_2_O emission, soil temperature, WFPS, and N_2_O-related gene numbers were analyzed by analysis of variance (ANOVA) for the least significant differences (LSD) at *p* < 0.05 level. Differences in cumulative N_2_O emission, soil temperature, WFPS, and N_2_O-related gene numbers between black plastic film mulching treatment and non-mulched treatment were analyzed by ANOVA for LSD at *p* < 0.05 level. The above statistical analyses were evaluated by SPSS (IBM SPSS statics version 24.0, SPSS Inc., Chicago, IL, USA). Graph drawing and regression models of soil N_2_O responding to soil abiotic and biotic parameters were performed by Origin and R language.

## 3. Results

### 3.1. N_2_O Flux and Soil Temperature

**Figure 1:**
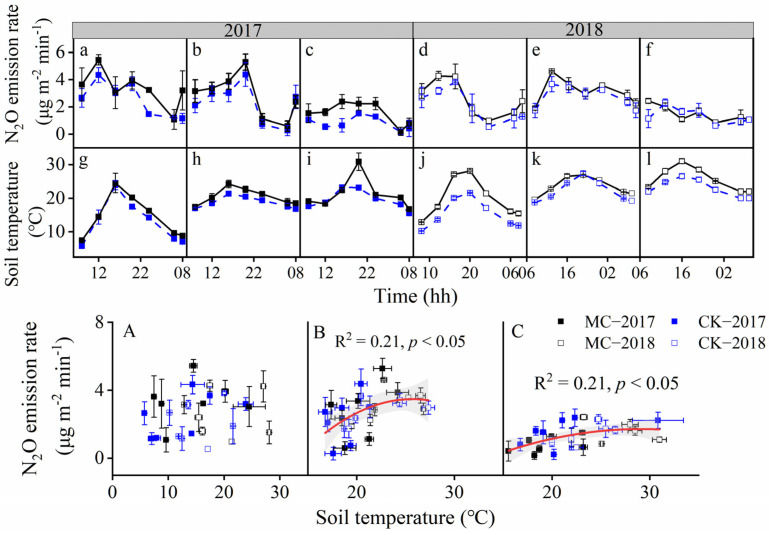
Diurnal variation of N_2_O emission rates and soil temperatures on three typical days at early (**a**,**d**,**g**,**j**), middle (**b**,**e**,**h**,**k**), and later (**c**,**f**,**i**,**l**) growth stage, and relationship between N_2_O emission rate with soil temperature at early (**A**), middle (**B**), and later (**C**) growth stage. The date of the three growth stages is introduced in Supplementary Methods. MC and CK are abbreviations for treatments with or without mulching, respectively. Vertical bars are the standard error of the mean (*n* = 3).

Nitrous oxide fluxes first increased and then decreased for both treatments from 8:00 a.m. to 8:00 a.m. on another day (Figure 1a–f). MC treatment significantly (*p* < 0.05) affected daily average N_2_O fluxes on all typical days, and significantly increased daily average N_2_O fluxes by 17–100% (Table S4).

Diurnal soil temperatures on typical days changed by a single-peak pattern (Figure 1g–l). MC treatment significantly (*p* < 0.05) promoted soil temperatures on typical days and significantly increased daily average N_2_O fluxes by 1.8–2.7 °C (Table S4).

There is a quadratic relationship between N_2_O emission rates and soil temperatures at the middle and later growth stages (Figure 1B,C). The N_2_O emission rate reached the greatest at about 25 and 29 °C at the middle and later growth stage, respectively. No significant relationship between N_2_O flux and soil temperature was found at the early growth stage (Figure 1A).

### 3.2. Soil N_2_O Flux Variation between Two Irrigation Events

**Figure 2:**
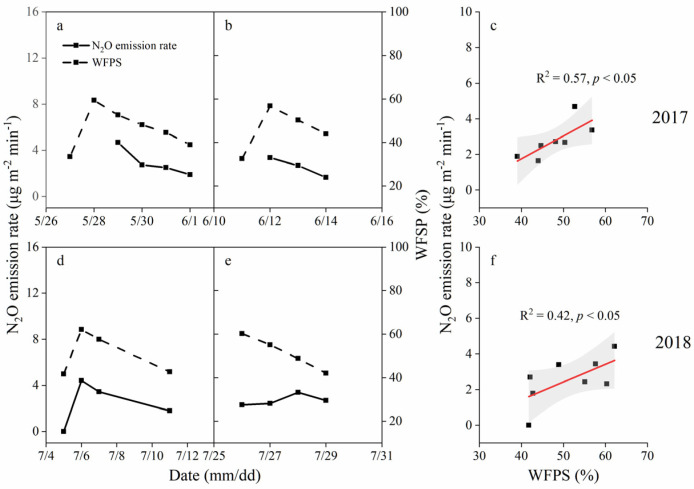
Variation of N_2_O emission rates and WFPS after irrigation under black plastic film mulching treatment in 2017 (**a**,**b**) and 2018 (**d**,**e**), and linear correlation between N_2_O emission rates and WFPS in 2017 and 2018 (**c**,**f**).

Soil N_2_O fluxes changed with a decreasing trend after the irrigation event except fluxes measured on 26–30 July (Figure 2). N_2_O fluxes from 26 to 30 July changed with a flat trend.

WFPS reached the field capacity after the irrigation event and declined to about 70% (*v/v*) of the field capacity before the next irrigation event (Figure 2 and Figure S5). Meanwhile, WFPS in MC treatment declined slower than in CK treatment after irrigation (Figure S5).

Soil N_2_O flux had a positive linear correlation (*p* < 0.05) with WFPS (Figure 2c,f). Fluxes of N_2_O increased with the increase of WFPS when WFPS changed within a range of 39–62%.

### 3.3. Soil Gas Variation

**Figure 3:**
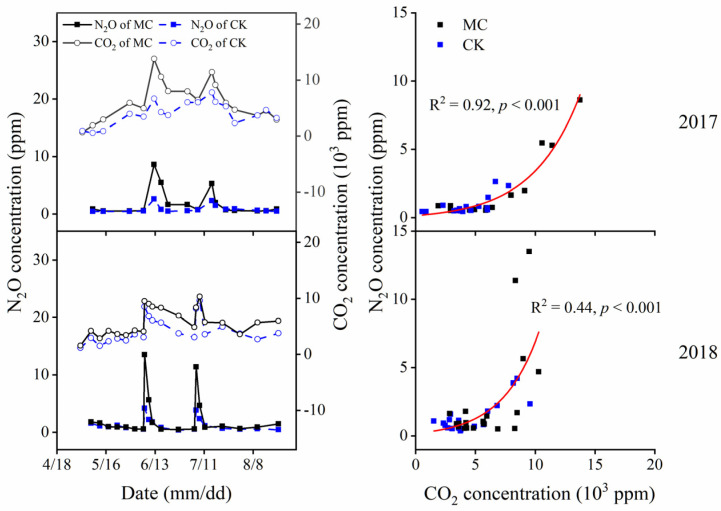
Variation of soil CO_2_ and N_2_O concentrations in 2017 and 2018 and the relationship between soil CO_2_ and N_2_O concentrations.

Soil CO_2_ showed a firstly increasing and then decreasing pattern with two peaks during the experiments (Figure 3). Topdressing brought about a small CO_2_ peak in both experimental years. MC treatment had higher soil CO_2_ than CK treatment. Across two years, average soil CO_2_ concentrations were 5.3–5.9 × 10^3^ and 3.5–4.0 × 10^3^ ppm for MC and CK, respectively.

Soil N_2_O concentration variation for MC and CK treatments were similar during the experiment, with clear patterns of increasing occurring following the topdressing and decreasing after several days after topdressing (Figure 3). MC treatment had the higher average soil N_2_O concentrations than CK treatment, being 1.7 (MC) and 1.0 (CK) ppm in 2017 and 3.3 (MC) and 3.1 (CK) ppm in 2018, respectively.

Soil N_2_O concentrations increased as soil CO_2_ concentrations increased, and this response was best fitted by an exponential model (Figure 3). Soil CO_2_ could explain 44–92% of the variance in soil N_2_O concentrations regarding the correlations between soil C_2_O and soil N_2_O (Figure 3).

### 3.4. Soil N_2_O-Related Microbial Genes

**Figure 4:**
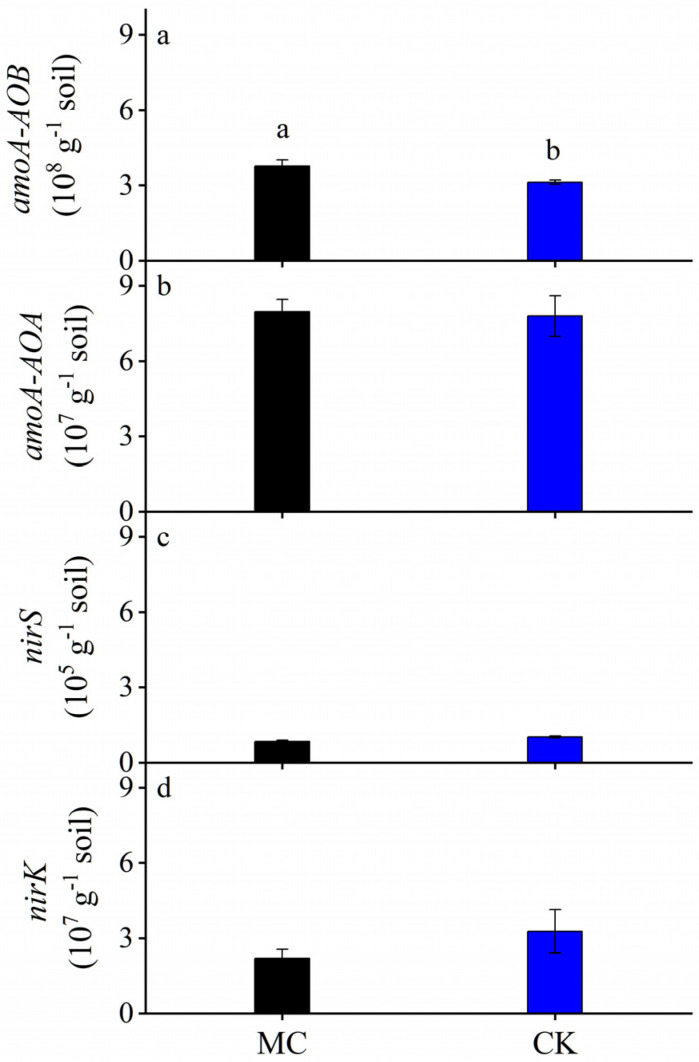
Soil N_2_Orelated gene abundances at the end of the experiment in 2018. Different letters above each bar indicate a significant difference between black plastic film mulching (MC) and non-mulched (CK) treatment at *p* < 0.05.

Black plastic film mulching significantly affected *amoA*-*AOB* (Figure 4 and Table 1). The abundance of the *amoA*-*AOB* gene was 3.76 × 10^8^ and 3.12 × 10^8^ copies per gram of soil for MC and CK treatment, respectively. The abundance of *amoA*-*AOB* increased by 21% under MC treatment, compared with the value under CK treatment. No significant difference in the abundance of *amoA*-*AOA*, *nirS*, and *nirK* genes between MC and CK treatment.

### 3.5. Seasonal N_2_O Fluxes

**Figure 5:**
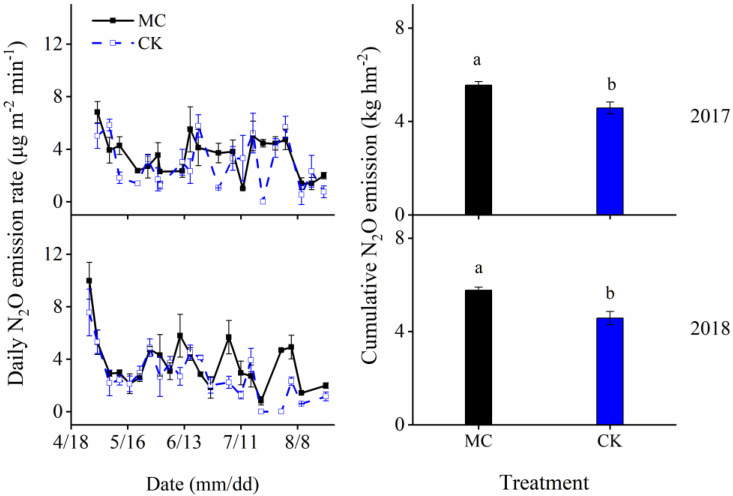
Daily N_2_O emission rates and cumulative N_2_O emissions under the soil with (MC) and without (CK) black plastic film mulching. Different letters above each bar indicate a significant difference between MC and CK at *p* < 0.05. Values of the columns and standard errors are shown in Table 1. Vertical bars are the standard error of the mean (*n* = 3).

Fluxes of N_2_O changed with fluctuations, and MC treatment had higher N_2_O fluxes than CK during the study periods (Figure 5). MC treatment significantly (*p* < 0.05) affected cumulative N_2_O fluxes (Figure 5 and Table 1). Over two study years, MC treatment increased cumulative N_2_O emission by 21–26%.

**Figure 6:**
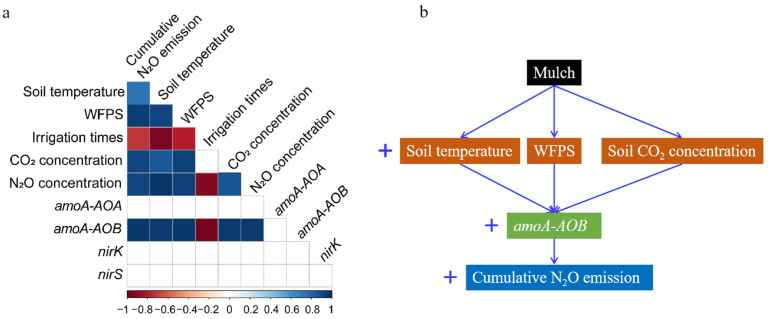
Correlations of cumulative N_2_O emission with soil temperature, WFPS, irrigation times, soil CO_2_ concentrations, soil N_2_O concentrations, and abundance of *amoAAOA*, *amoAAOB*, *nirK*, and *nirS* genes (**a**). Conceptual diagrams showing regulators and controls of black plastic film mulching effect on cumulative N_2_O emission (**b**).

The cumulative N_2_O flux was positively correlated with soil temperature, soil WFPS, soil CO_2_ concentrations, soil N_2_O concentrations, and *amoA*-*AOB* gene abundance, while negatively correlated with irrigation times (Figure 6).

## 4. Discussion

The effect of black plastic film mulching (MC) treatment on N_2_O emission differs based on previous studies. Studies indicated that MC treatment increased [23], reduced [10,13], or did not affect [24,25] N_2_O emissions compared with non-mulched treatment. The influence of MC treatment on soil environmental conditions varying with the experimental sites could be the reason for the controversial results. Zhao [10] suggested MC treatment reduced N_2_O emissions in the hot pepper season owing to lower soil moisture. Reduction of the available mineral N due to the enhanced N uptake of plants would be another reason for the limiting of nitrification and denitrification processes under black plastic film mulching [13]. On the other hand, N_2_O emissions may not differ between the mulched and non-mulched fields, mainly because film mulching did not affect the soil temperature due to the cooling effect of irrigation [25]. While consistent with Yu [13] and Gao [26], our results showed that MC treatment significantly increased N_2_O emissions, mainly through regulating biotic (*amoA*-*AOB*) and abiotic (soil temperature, soil moisture, and soil CO_2_ concentrations) parameters (Figure 6).

In this study, N_2_O emissions had a quadratic relationship with soil temperatures during the 24-h measurement (Figure 1B,C), and cumulative N_2_O emissions also showed a positive relationship with average soil temperatures across two experimental years (Table 1, Figure S3 and Figure 6). Our results indicated that soil temperature was a vital abiotic regulator of soil N_2_O emission. The failure to find a correlation between N_2_O flux and soil temperature at the early growth stage (Figure 1A) may be attributed to the lower sensitivity of N_2_O-related microorganisms to soil temperature when sufficient nutrients in soils at the early growth stage [27]. Zhao [10] indicated that N_2_O emissions could increase with the increase of soil temperature (from 5 to 32 °C), while we found N_2_O emissions might decrease when soil temperature was above 25–29 °C at middle and later growth stages (Figure 1B,C). Similar results had been reported that N_2_O emissions would be inhibited when soil temperature was above 30 °C [11]. Soil N_2_O emissions could be potentially inhibited by black plastic film mulching for extremely high temperatures. Moreover, from the perspective of cumulative N_2_O emissions during the whole growing season, the positive effect outweighed the negative one of mulching–induced warming on cumulative N_2_O emissions. The positive linear correlation between cumulative N_2_O emission and average soil temperature (Figure 6) could support this inference.

Soil moisture was another primary factor affecting N_2_O emissions. MC treatment could significantly increase soil moisture, which has been reported by lots of studies [8,21]. We also found soil N_2_O emissions had a positive correlation with WFPS (from 39% to 62%) (Figure 2), which was consistent with that of Zhao [10]. Zhao indicated that the N_2_O emission rate was exponentially correlated with WFPS when WFPS varied from 20% to 80% [10]. Results also showed that average WFPS had a positive linear correlation with cumulative N_2_O emissions, which supports our inference that higher soil moisture under MC would be conducive to N_2_O production. On the other hand, soil moisture was observed to decrease more rapidly after irrigation under CK treatment (Figure S5), which indicated that soil humidity would retain at a higher level for N_2_O production for a longer time under MC treatment.

As reported that the denitrification process produced the greatest N_2_O at 70 to 90% WFPS [28], the nitrification process might domain N_2_O productions in this study (Figure S4). Ammonia oxidation is the first and rate-limiting step in nitrification, and it is catalyzed by an ammonia monooxygenase (AMO) encoded in gene *amoA* of *AOA* and *AOB* [29]. Although several studies have reported *amoA*-*AOA* to play an important role in nitrification [29,30], results in this study showed that MC treatment only significantly enhanced the abundance of *amoA*-*AOB* (Table 1 and Figure 4), and *amoA*-*AOB* abundances were positively correlated with cumulative N_2_O emissions (Figure 6). It was *AOB*, not *AOA* or other denitrification-related microorganisms, that induced the N_2_O emission under MC treatment. Previous studies also indicated that *amoA*-*AOB*-induced nitrification was regarded as the key pathway for N_2_O production [31]. Higher *amoA*-*AOB*–dependent N_2_O product stoichiometry than *amoA*-*AOA* was likely due to the incomplete NH_2_OH oxidation and nitrifier denitrification, as previously observed in neutral/alkaline soils [31,32]. Since some *amoA*-*AOB* had genes encoding a canonical hydroxylamine dehydrogenase and NO reductase and thus could conduct nitrifier denitrification, they played a more important role in these alkaline mulched soils (Figure 6). Notably, CO_2_ concentrations under MC treatment were higher than those under CK, which could be attributed to that MC treatment enhancing soil microbes’ activity [33,34]. We found significant exponential correlations between soil N_2_O and CO_2_ concentrations (Figure 3), and cumulative N_2_O emissions were significantly correlated with soil CO_2_ concentrations (Figure 6). As previous studies mentioned, elevated atmospheric CO_2_ increased soil nitrification rate and shifted ammonia-oxidizing community abundance and structure [35,36,37]. Elevated CO_2_ could both significantly shift *amoA*-*AOB* and *amoA*-*AOA* communities, and *amoA*-*AOB* was more sensitive to the rising CO_2_ concentration [35]. We infer that high CO_2_ concentration under black plastic film mulching might be conducive to the growth of *amoA*-*AOB* and promote N_2_O emissions.

From the above analyses, it can be seen that the MC treatment plays a primary role in regulating N_2_O emissions. This study showed a negative impact of MC treatment on greenhouse gas mitigation. On the other hand, studies have reported a significant positive effect of MC treatment on potato harvest [8]. Therefore, further research should be devoted to examining the impacts of MC treatment on both potato harvests and N_2_O emissions.

## 5. Conclusions

This study used the static chamber-gas chromatography method to evaluate the effect of black plastic film mulch treatment on soil N_2_O emission in the arid potato field. Our results demonstrated that black plastic film mulch treatment can significantly increase soil cumulative N_2_O emission. We identified that black plastic film mulching promoted soil N_2_O emissions mainly by altering abiotic parameters including increasing soil temperatures, soil moisture, and soil CO_2_ concentrations. We also highlighted that it was *amoA-AOB*, but not other N_2_O-related microorganisms that regulated N_2_O emission under plastic film mulching. Further research should be devoted to examining the impacts of black plastic film mulch treatment on greenhouse gas emissions apart from food production.

## Figures and Tables

**Table 1:** Cumulative N_2_O emissions, soil temperatures, WFPS, and soil N_2_O-related gene abundances as affected by black plastic film mulching treatment.

Year	Parameter	MC ^1^	CK	ANOVA
2017	Cumulative N_2_O emission (kg hm^−2^)	5.56 ± 0.15 a	4.58 ± 0.25 b	*
	Soil temperature (°C)	20.7 ± 0.5 a	18.2 ± 0.3 b	*
	WFPS (%)	48.4 ± 0.4	46.1 ± 0.2	*
2018	Cumulative N_2_O emission (kg hm^−2^)	5.77 ± 0.13 a	4.57 ± 0.28 b	*
	Soil temperature (°C)	21.6 ± 0.0 a	19.6 ± 0.3 b	*
	WFPS (%)	48.4 ± 0.1	46.7 ± 0.1	*
	*amoA*-*AOA* (10^7^ g^−1^ soil)	7.96 ± 0.50	7.80 ± 0.85	ns
	*amoA*-*AOB* (10^8^ g^−1^ soil)	3.76 ± 0.20 a	3.12 ± 0.04 b	*
	*nirK* (10^5^ g^−1^ soil)	7.34 ± 0.07	7.51 ± 0.12	ns
	*nirS* (10^7^ g^−1^ soil)	4.91 ± 0.03	5.00 ± 0.02	ns

^1^ Note: MC and CK are abbreviations for treatments with or without mulching, respectively. Different letters near the values indicate a significant difference between MC and CK at *p* < 0.05. Values are means ± SE (*n* = 3). *: difference between treatments was significant (*p* < 0.05); ns: there was no significant difference.

## Data Availability

The data presented in this study are available on request from the corresponding author.

## Supplementary Materials

The following supporting information can be downloaded at: https://www.mdpi.com/article/10.3390/ijerph192316030/s1, Supplementary Methods; Supplementary Results; Table S1: Soil properties of the top 20 cm soil before experiment; Table S2: Fertilization and agronomic practice in this study. Table S3: Primers and thermal cycling conditions used for quantitative PCR; Table S4: Daily average N_2_O fluxes and daily average soil temperatures as affected by black plastic film mulching treatment on typical days in 2017 and 2018; Figure S1: Dynamics of air temperature and precipitation during the experiment; Figure S2: Schematic diagram of the soil-air equilibration tube; Figure S3: Daily average soil temperature at 10 cm-depth of soils covered with (MC) or without (CK) black plastic film mulch; Figure S4: Variation of WFPS at 20 cm-depth of soils covered with (MC) or without (CK) black plastic film mulch; Figure S5: WFPS at 20 cm-depth of soils covered with (MC) or without (CK) black plastic film mulch between two irrigation events. References [38,39,40,41,42,43] are cited in Supplementary Materials.
