# Supplementary material for: Black Plastic Film Mulching Increases Soil Nitrous Oxide Emissions in Arid Potato Fields
Source: Int J Environ Res Public Health. 2022 Nov 30;19(23):16030. doi: 10.3390/ijerph192316030 (PMC9736522; doi:10.3390/ijerph192316030)
Supplement: Supplementary file 1 [file ijerph-19-16030-s001.zip › ijerph-2047066-supplementary.pdf]

Supplementary Information

Supplementary methods

N_2O flux sampling

The flux of N_2O was measured by a static opaque chamber-gas chromatography method. The upper chamber (80 cm long, 40 cm wide, and 70 cm high) was made of polymethyl methacrylate. A heat insulation foam sponge (1 cm thick) wrapped the upper chamber. A 9 cm diameter fan was installed, and two holes with a diameter of 6.5 mm were fitted for a thermometer and a silicone gas extraction tube at the top side of the upper chamber. The thermometer was inserted 15 cm into the chamber, and the silicone tube was inserted 35 cm into the chamber. During the measurement of flux, the upper chambers were placed on pre-installed stainless steel frames. Each frame (80 cm long, 40 cm wide, and 35 cm high) was inserted 5 cm into the soil of one ridge in each plot and included soil and two crops. A water trough with a 3 cm width and 3 cm height connected the upper chamber and frame for an airtight seal. Five gas samples were collected to calculate N_2O flux between 8:00 am and 10:00 am after chamber closure, at a time interval of 4 minutes about every 5 days. Gas samples were then kept in pre-vacuumed plastic gasbags (Dalian Pulaite gas packing Co., Ltd., Dalian, China) and analyzed for the N_2O concentration using a gas chromatograph.

Diurnal N_2O flux variations were measured to identify the effect of different treatments on N_2O flux by alternating soil temperature. Fluxes of N_2O were measured on 4th-5th May (early growth stage), 4th-5th July (middle growth stage), and 2nd-3rd August (later growth stage) in 2017, and 27th-28th May (early growth stage), 21st-22nd June (middle growth stage) and 31st-1st august (later growth stage) in 2018. All the sampling days were days before the irrigation events. Seven flux samples were taken at 8:00, 12:00, 16:00, 20:00, 24:00, 6:00, and 8:00.

Fluxes between two irrigation events were measured to identify the relationship between N_2O flux and soil moisture. Gases samples were collected between 8:00 am and 9:00 am on 29th May-1st June and 12th-14th June in 2017 and 5th-11th July and 26th-29th July in 2018.

Soil gas (CO_2 and N_2O) sampling

One plot of each treatment was chosen for gas sampling. One soil-air equilibration sampler was installed vertically in the soil to a soil depth of 15-20 cm (Fig. S2). The soil-air equilibration sampler was modified from Song [1] and consisted of an un-plasticized polyvinylchloride (PVC-U) tube, a PVC plug blocking the top of the PVC-U tube, and a silicone tube inserted into the PVC-U tube through the PVC plug. The PVC-U tube and PVC plug had internal diameters of 2.5 cm. Every PVC-U tube had eight small holes with a diameter of 2 mm, and all the holes were at the tube bottom. A nylon gauze wrapped the bottom of the PVC-U tube to stop the soil from clogging the tube (Fig. S2). We extracted 20 mL of gas samples between 10:00 and 10:30 after soil N_2O flux sampling, using a syringe through a three-way stopcock connecting with the silicon tube. Gas samples were then kept in pre-vacuumed plastic gasbags (Dalian Pulaite gas packing Co., Ltd., Dalian, China). The Three-way stopcock of the sampler was closed on non-sampling days to avoid the connection of soil air to atmospheric air.

Supplementary results

Soil temperature

Soil temperatures changed with substantial variations during the study periods in both years (Fig, S3). Soil temperatures for MC treatment ranged from 7-38 and 8-36 °C with average temperatures of 21.1 and 21.7 °C in 2017 and 2018. Soil temperatures for CK treatment ranged from 5-32 and 6-33 °C with average temperatures of 18.4 and 19.4 °C in 2017 and 2018. Over two

years, average soil temperatures for MC treatment were 1.8-2.7 °C higher than CK treatment.

Water-filled pore space

Water-filled pore space (WFPS) changed with fluctuations and ranged from about 70% (v/v) field capacity to field capacity for all treatments during the study periods in both years (Fig. S4). MC treatment significantly increased WFPS in both experimental years (Table 1). In 2017, the average WFPS for MC and CK treatment was 48.4%, and 46.1%, respectively. In 2018, the average WFPS for MC and CK treatment was 48.4, and 46.7%, respectively.

Table S1. Soil properties of the top 20 cm soil before the experiment

Year	NO_3^--N (mg/kg) [1]	NH_4^+-N (mg/kg)	SOC (g/kg)
2017	4.2 ± 2.2	4.2 ± 2.2	4.2 ± 2.2
2018	4.5 ± 0.2	4.5 ± 0.2	4.5 ± 0.2

[1] Note: Soil properties were determined according to Bao [2]. Values are means ± SE (*n = 6*)

Table S2. Fertilization and agronomic practice in this study

Fertilization and agronomic practice[1]	2017	2018
Basal fertilization	4/16	4/3
Sowing	4/26	4/17
Topdressing	6/11	6/6
Topdressing	7/15	7/5
Harvesting	8/26	8/28

[1]Notes: plots were fertilized with inorganic N (Urea), P (calcium super-phosphate), and K (potassium sulfate) at rates of 200 (2017) or 220 (2018) kg N ha^{-1}, 180 kg K ha^{-1} and 150 kg P ha^{-1}, respectively. Total P and 40% of total N and K were broadcast as basal fertilizer. The remaining 60% of the total N and K were fertilized separately through a drip irrigation system.

Table S3. Primers and thermal cycling conditions used for quantitative PCR

Gene	Primer	Sequence 5'-3'	Thermal cycling conditions	Reference
amoA-AOA	Arch-amoAR	GCGGCCATCCATCTGTATGT	95 °C for 1 min - × 1 cycle; 95 °C for 20 s, 55 °C for 30 s,	[3]
	Arch-amoAF	STAATGGTCTGGCTTAGACG	72 °C for 30 s - × 40 cycles; 72 °C for 8min	
amoA-AOB	amoA-1F	GGGGTTTCTACTGGTGGT	95 °C for 1 min - × 1 cycle; 95 °C for 20 s, 57 °C for 30 s,	[4]
	amoA-2R	CCCCTCKGSAAAGCCTTCTTC	72 °C for 30 s - × 40 cycles; 72 °C for 8min	
nirS	nirS-cd3aF	GTSAACGTSAAGGARACSGG	94 °C for 2 min - × 1 cycle; 94 °C for 30 s, 51 °C for 1	[5]
	nirS-R3cd	GASTTCGGRTGSGTCTTGA	min, 72 °C for 1 min - × 35 cycles; 72 °C for 10min	
nirK	nirK-FlaCu	ATCATGGTSCTGCCGCG	94 °C for 3 min - × 1 cycle; 94 °C for 30 s, 57 °C for 1	[6]
	nirK-R3Cu-GCb	GCCTCGATCAGRTTGTGGTT	min, 73 °C for 1 min - × 35 cycles; 75 °C for 10min	

Table S4 Daily average N_2O fluxes and daily average soil temperatures as affected by black plastic film mulching treatment on typical days in 2017 and 2018

Year	Treatment	Early stage		Middle stage		Later stage	
		Daily average N_2O flux	Daily average soil temperature	Daily average N_2O flux	Daily average soil temperature	Daily average N_2O flux	Daily average soil temperature
2017	MC[1]	3.4 ± 0.2 a	14.5 ± 0.7 a	2.7 ± 0.0 a	20.4 ± 0.2 a	1.6 ± 0.2 a	21.2 ± 0.4 a
	CK	2.7 ± 0.0 b	13.0 ± 0.2 b	2.3 ± 0.1 b	18.7 ± 0.3 b	0.8 ± 0.1 b	19.5 ± 0.0 b
2018	MC	2.6 ± 0.2 b	19.8 ± 0.1 b	3.1 ± 0.1 a	23.5 ± 0.2 a	1.0 ± 0.0 a	25.7 ± 0.2 a
	CK	2.1 ± 0.1 c	15.3 ± 0.1 c	2.5 ± 0.2 b	22.1 ± 0.0 b	0.7 ± 0.2 b	23.0 ± 0.3 c

[1]Note: MC and CK are abbreviations for treatments with or without black plastic film mulching, respectively. The sampling time was on 4th-5th May (early growth stage, a and g), 4th-5th July (middle growth stage, b, and h), and 2nd-3rd August (later growth stage, c and i) in 2017, and on 27th-28th May (early growth stage, d, and j), 21st-22nd June (middle growth stage, e, and k), and 31st July-1st August (later growth stage, f, and l) in 2018. Different letters (a, b and c) behind numbers mean the differences among values for different treatments were significant at $p < 0.05$ level. Values with the same letter were statistically similar at $p < 0.05$ level. Values are means ± SE ($n = 3$)

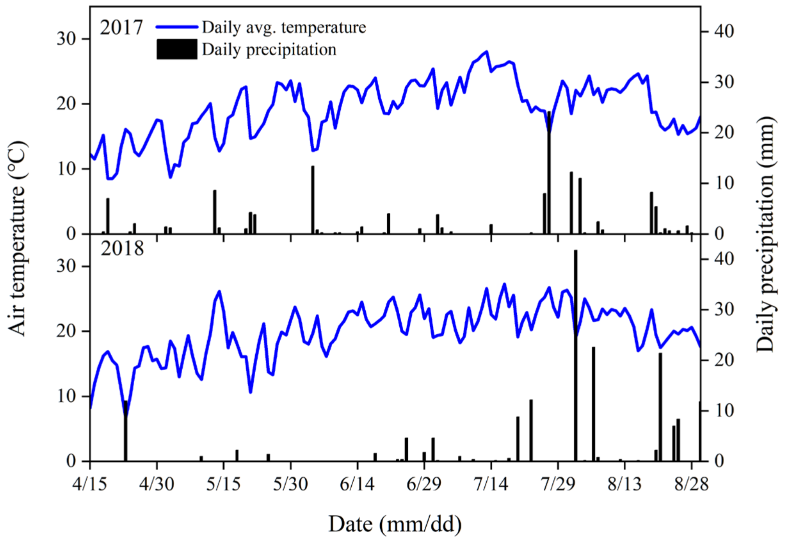

Figure S1 Dynamics of air temperature and precipitation during the experiment

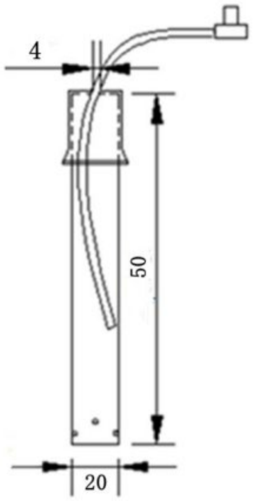

Figure S2 Schematic diagram of the soil-air equilibration tube

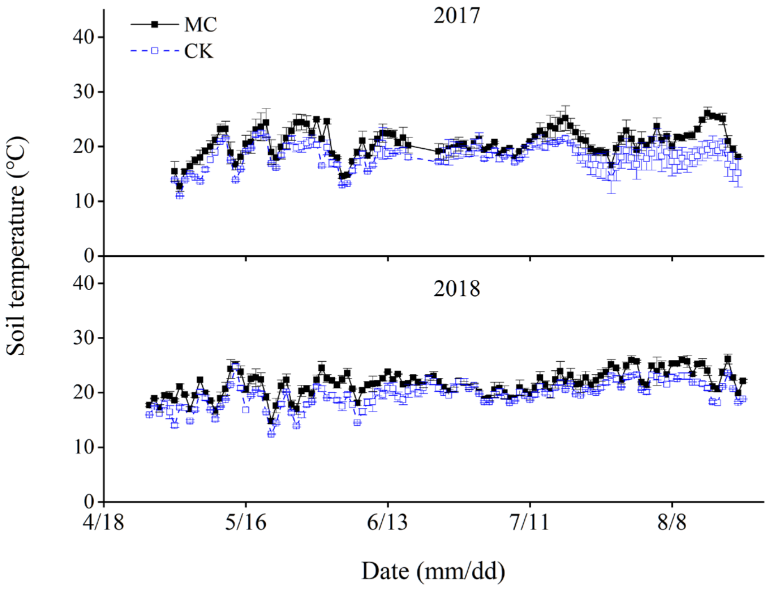

Figure S3 Daily average soil temperature at 10 cm-depth of soils covered with (MC) or without (CK) black plastic film mulch

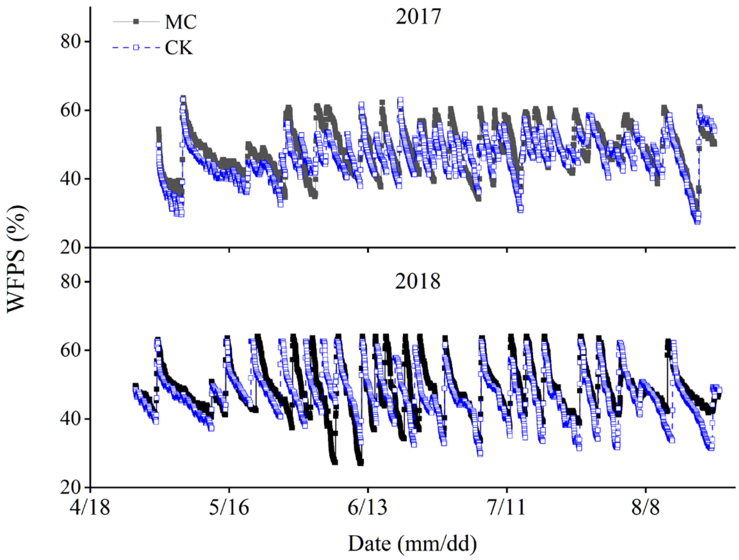

Figure S4 Variation of WFPS at 20 cm-depth of soils covered with (MC) or without (CK) black plastic film mulch

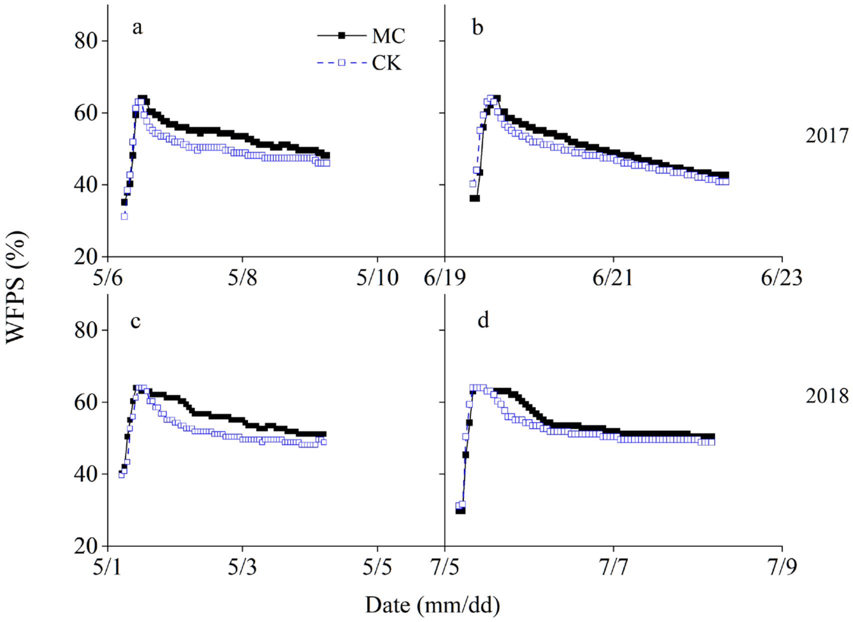

Figure S5 WFPS at 20 cm-depth of soils covered with (MC) or without (CK) black plastic film mulch between two irrigation events.

References:

1. Song, X.; Ju, X.; Topp, C.F.E.; Rees, R.M. Oxygen Regulates Nitrous Oxide Production Directly in Agricultural Soils. *Environ Sci Technol* **2019**, 53, 12539-12547, doi:10.1021/acs.est.9b03089.
2. Shudan, B. *Soil Agro-Chemistry Analysis*. China Agriculture Press: Beijing, 2005; p.
3. Francis, C.A.; Roberts, K.J.; Beman, J.M.; Santoro, A.E.; Oakley, B.B. Ubiquity and diversity of ammonia-oxidizing archaea in water columns and sediments of the ocean. *P Natl Acad Sci Usa* **2005**, 102, 14683-14688.
4. Rotthauwe, J.H.; Witzel, K.P.; Liesack, W. The ammonia monooxygenase structural gene amoA as a functional marker: molecular fine-scale analysis of natural ammonia-oxidizing populations. *Appl Environ Microb* **1997**, 63, 4704-4712, doi:10.1128/AEM.63.12.4704-4712.1997.
5. ThrobÃ Ck, I.N.; Enwall, K.; Jarvis, Ã.S.; Hallin, S. Reassessing PCR primers targeting nirS, nirK and nosZ genes for community surveys of denitrifying bacteria with DGGE. *Fems Microbiol Ecol* **2004**, 49, 401-417, doi:10.1016/j.femsec.2004.04.011.
6. Hallin, S.; Lindgren, P. PCR Detection of Genes Encoding Nitrite Reductase in Denitrifying Bacteria. *Appl Environ Microb* **1999**, 65, 1652-1657, doi:10.1128/AEM.65.4.1652-1657.1999.
